# Combination of DXA and BIS Predicts Jump Power Better Than Traditional Measures of Sarcopenia

**DOI:** 10.1002/jbm4.10527

**Published:** 2021-07-02

**Authors:** Benjamin Rush, Neil Binkley, Diane Krueger, Yosuke Yamada, Adam J Kuchnia

**Affiliations:** ^1^ Department of Nutritional Sciences University of Wisconsin‐Madison Madison WI USA; ^2^ University of Wisconsin Osteoporosis Clinical Research Program Madison WI USA; ^3^ Department of Physical Activity, National Institute of Health and Nutrition National Institutes of Biomedical Innovation, Health and Nutrition Tokyo Japan

**Keywords:** BIOIMPEDANCE SPECTROSCOPY (BIS), DUAL‐ENERGY X‐RAY ABSORPTIOMETRY (DXA), MUSCLE FUNCTION, MUSCLE QUALITY, SARCOPENIA

## Abstract

Traditional diagnostic criteria for sarcopenia use dual‐energy X‐ray absorptiometry (DXA)‐measured appendicular lean mass (ALM), normalized to height (ALM/ht^2^) or body mass index (ALM/BMI) to define low muscle mass. However, muscle function declines with aging before the loss of muscle mass is detected by ALM. This is likely due, in part, to qualitative muscle changes such as extracellular and intracellular fluid compartment shifts uncaptured by DXA. We propose combining bioimpedance spectroscopy (BIS), which estimates extracellular and intracellular compartment volume, with DXA to more accurately predict muscle function. This combination may help incorporate muscle quality, thereby improving sarcopenia diagnosis. We cross‐sectionally analyzed data from 248 Black and White participants aged 25 to 75 years from the Midlife in the United States Refresher Cohort. We proposed two novel muscle measures: ALM corrected by the BIS‐derived whole‐body extracellular to intracellular fluid ratio (E/I) and leg lean mass (LLM) corrected by leg‐specific E/I, creating (ALM/(E/I)_W_) and (LLM/(E/I)_L_), respectively. We compared the associations of traditional muscle measures, ALM/(E/I)_W_, and LLM/(E/I)_L_, with grip strength and lower limb power using jumping mechanography. LLM/(E/I)_L_ explained jump power best at *R* ^2^ = 0.803 compared with ALM/(E/I)_W_ (*p* < 0.0001) and all other measures. ALM/(E/I)_W_ explained jump power second best (*R* ^2^ = 0.759) but not significantly better than traditional muscle measures. No muscle measure performed better than covariates when predicting handgrip strength. LLM/(E/I)_L_ outperformed ALM/ht^2^ and ALM/BMI when predicting jump power. We propose LLM/(E/I)_L_ is a powerful and clinically relevant method that accounts for muscle quality. © 2021 The Authors. *JBMR Plus* published by Wiley Periodicals LLC on behalf of American Society for Bone and Mineral Research.

## Graphical abstract

We show that the combination of DXA and BIS derived leg lean mass corrected by the ratio of extracellular fluid in intracellular fluid better predicts jump power than appendicular lean mass adjusted by height or body mass index. Appendicular lean mass or leg lean mass corrected by height, body mass index, or the ratio of intracellular fluid to extracellular fluid did not predict handgrip strength better than covariates only.

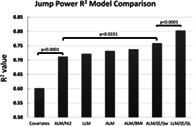

## 1 Introduction

Sarcopenia, the progressive loss of muscle mass and function, puts older individuals at higher risk of immobility, quality of life decline, and mortality.^(^ ^1^, ^2^, ^3^, ^4^, ^5^, ^6^ ^)^ In addition, sarcopenia increases health care cost.^(^ ^7^, ^8^ ^)^ Although sarcopenia is becoming a major public health burden, it is clinically unacknowledged and underdiagnosed, thereby impairing care of older adults.^(^ ^5^ ^)^

According to the updated 2019 European Working Group on Sarcopenia in Older Adults guidelines, the first indications of sarcopenia are low muscle strength and function later confirmed by low muscle mass measurement.^(^ ^1^ ^)^ Low muscle function, often assessed by handgrip strength, is traditionally associated with low muscle mass estimated by appendicular lean mass (ALM).^(^ ^9^ ^)^ ALM is the sum of lean mass in the arms and legs typically measured by dual‐energy X‐ray absorptiometry (DXA) to estimate appendicular muscle mass.^(^ ^1^, ^9^ ^)^ To account for variation in the size of individuals, ALM is normalized by height (ALM/ht^2^) or body mass index (BMI; ALM/BMI).^(^ ^1^, ^9^ ^)^

Although frequently used in sarcopenia diagnosis, DXA is limited in that it measures three compartments: bone, fat, and all others (generally referred to as lean mass, which includes muscle tissue).^(^ ^10^ ^)^ Muscle tissue contains fluid compartments, which are major contributors to ALM.^(^ ^11^ ^)^ As people age, the fluid compartments within muscle tissue change with the preservation or slight increase of extracellular fluid (ECF) but a decline in intracellular fluid (ICF).^(^ ^11^, ^12^ ^)^ However, this loss of ICF and conservation of ECF with aging results in a higher ECF to ICF (E/I) ratio within muscle, which is significant because reduced ICF is associated with the loss of muscle function with age.^(^ ^11^, ^13^, ^14^ ^)^ The retention of ECF is also thought to mask true muscle atrophy when measuring ALM because DXA cannot distinguish between ICF and ECF and instead categorizes both as lean mass.^(^ ^13^ ^)^

Conversely, bioimpedance spectroscopy (BIS) can distinguish between ICF and ECF.^(^ ^14^ ^)^ Furthermore, BIS‐derived ICF and ECF can be segmentally assessed in the arms and legs, yielding both whole‐body and regional ratios of ICF to ECF.^(^ ^11^ ^)^ The decline in leg‐specific BIS measures are more strongly associated with age than DXA‐derived leg lean mass and thus may prove useful for assessing muscle mass.^(^ ^11^ ^)^ However, BIS becomes less accurate at higher or lower BMI as the assumptions used for body size modeling do not account for all sizes.^(^ ^12^ ^)^ To account for the variation from BMI, BIS‐derived ICF and ECF can be adjusted for differences in BMI, yielding a corrected whole‐body E/I ratio ((E/I)_W_).^(^ ^12^ ^)^

To mediate the limitations of DXA and incorporate the benefits of BIS to predict muscle function, we propose novel measures using a combination of DXA and BIS, which incorporate both muscle mass and muscle quality. Muscle quality encapsulates the physiological and metabolic state of muscle tissue and is theorized to explain the loss of muscle function before the loss of muscle mass.^(^ ^15^, ^16^ ^)^ No consensus measures of muscle quality exist; however, fatty or connective tissue infiltration within muscle tissue have been proposed as measures of muscle quality.^(^ ^1^, ^17^, ^18^ ^)^ We propose BIS‐derived E/I as another measure of muscle quality.

In a previous study, we tested a novel measure of DXA‐derived ALM adjusted for BIS‐derived E/I against the traditional mass only measures ALM/ht^2^ and ALM/BMI. We showed that ALM/(E/I)_W_ is more highly associated with handgrip strength and jump power than ALM/ht^2^ and ALM/BMI in an older, mostly White cohort.^(^ ^16^ ^)^ Both handgrip strength and jump power are measurements of muscle function; however, jump power declines more with age than handgrip strength does with age, and thus is possibly a better method of assessing muscle function when diagnosing sarcopenia.^(^ ^19^ ^)^ Jump power assesses an individual's power generated from a dynamic, coordinated jump registered by a force plate using multiple muscle groups and is thought to be a more comprehensive muscle function test than handgrip strength.^(^ ^20^, ^21^ ^)^ Because both diminished lower leg strength and sarcopenia are associated with a higher risk of falls, predicting leg muscle function specifically may better determine those at higher risk of falls.^(^ ^22^, ^23^ ^)^ Therefore, we propose another measure of DXA‐derived leg muscle mass that incorporates a surrogate of muscle quality measured by adjusting leg lean mass (LLM) for the BIS‐derived, leg‐specific ratio of extracellular to intracellular fluid, or LLM/(E/I)_L_.

We hypothesize our novel measures of muscle, which incorporate a surrogate of muscle quality and muscle mass, are more associated with muscle function compared with traditional muscle mass‐based measures. The purpose of this study is to further explore ALM/(E/I)_W_ and LLM/(E/I)_L_ as improved measures of muscle by determining their associations with muscle function compared with ALM/ht^2^ and ALM/BMI in a larger, more diverse population, furthering our understanding of sarcopenia.

## 2 Materials and Methods

We tested our hypothesis in volunteers participating in the Midlife in the United States (MIDUS) Refresher cohort. MIDUS is a multisite, national prospective cohort study that began in 1995.^(^ ^24^ ^)^ Because of loss of follow‐up, the MIDUS Refresher cohort was established as a separate cohort and enrolled additional participants aged 25 to 75 years starting in 2011. The University of Wisconsin Health Sciences Institutional Review Board approved this study, and researchers conducted the study in compliance with global, national, and local regulations. We cross‐sectionally analyzed data from the first study visit from MIDUS Refresher participants who completed their study visit at the Osteoporosis Clinical Research Center in Madison, WI, as this was the only MIDUS site to conduct both DXA and BIS (*n* = 267). We further restricted our analysis to participants who identified their race as Black or White (*n* = 248) for an adequate statistical comparison between racial groups.

### 2.1 Demographic and anthropomorphic measures

Demographic data including race, age, and sex were collected during a phone interview. Height measured by calibrated Harpenden wall‐mounted stadiometer (Holtain, Crymych Wales, UK) and weight using a calibrated analog scale were collected during the research study visit. BMI was calculated using weight in kilograms divided by height in meters squared (kg/m^2^). We generated age categories because age was non‐normally distributed after transformation. The age categories were based on mean age quartiles and grouped from 25 to 40, 41 to 50, 51 to 61, and older than 61 years. Physical activity was assessed by self‐reported metabolic equivalent of tasks (METs). METs were calculated by multiplying the minutes of each exercise activity by intensity (6 METS for vigorous, 3 METs for moderate, and 1.1 METs for light) and by the frequency of each activity per week. Because of a non‐normal distribution in METS after transformation, physical activity was converted from continuous to dichotomous with a participant labeled “non‐sedentary” if that participant self‐reported METs greater than 0.

### 2.2 Muscle function measures

All muscle function measures were collected during the research study visit. Handgrip strength was measured using a Jamar handheld dynamometer (JLW Instruments, Chicago, IL, USA) and jump power was determined using a jump mechanography force plate (Leonardo, Novotec, Pforzheim, Germany) following established standard operating procedures. To obtain handgrip strength, we used each participant's maximum of three grip trials by their non‐dominant hand while sitting and flexing their elbow 90° according to established protocol.^(^ ^25^, ^26^ ^)^ To obtain jump power, each participant performed three countermovement jumps on a force plate; the jump with the maximal power from the highest jump was used according to established protocol.^(^ ^20^ ^)^ Leonardo software 4.2 calculated the maximum jump power. We defined jump power as maximum jump power because it captures a measurement of individuals not able to fully be airborne when jumping and is a measurement of the countermovements assessed by the force plate.^(^ ^27^ ^)^

### 2.3 Lean mass and quality measures

All muscle measures were collected during the research study visit. DXA measurements were acquired and analyzed using a Lunar iDXA densitometer with enCORE software v17 (GE Healthcare, Madison, WI, USA) by International Society for Clinical Densitometry–certified technologists following manufacturer recommendations. Scans were autoanalyzed by the software and manually adjusted when errors were identified. ALM is the sum of lean tissue mass from each individual arm and leg. Likewise, LLM is the sum of the lean mass in both legs. We calculated ALM/ht^2^ and ALM/BMI by dividing ALM by height squared or BMI, respectively.

Participant BIS exams were performed using an ImpediMed SFB7 (Impedimed, Pinkenba, Australia). For whole‐body measurement, the current injecting electrodes were placed on the left side of the body on the dorsal surface of the hand and foot proximal to the metacarpal‐phalangeal and metatarsal‐phalangeal joints, respectively. The sensing electrodes were placed medially between the distal prominences of the radius and ulna at left wrist and between the medial and lateral malleoli at the left ankle.^(^ ^16^ ^)^ Left leg only measurements were performed by placing the current injecting electrodes on the left side of the body on the dorsal surface of the hand and foot identical to the whole‐body measurement. The sensing electrodes were placed on each ankle.^(^ ^11^, ^28^ ^)^ These measurements yielded whole‐body and left leg ICW and ECW through Impedimed proprietary algorithms using Hanai's mixture conductivity theory previously described elsewhere.^(^ ^12^ ^)^

To generate our ALM/(E/I)_W_ measure, we normalized ALM by the whole‐body ratio of extracellular to intracellular fluid corrected by BMI ((E/I)_W_). Further details about the calculation of ALM/(E/I)_W_ are described elsewhere.^(^ ^13^, ^16^ ^)^ To produce our LLM/(E/I)_L_ measure, we adjusted LLM by the leg‐specific ratio of left leg extracellular fluid to left leg intracellular fluid. We assumed the ratio of left leg extracellular to left leg intracellular fluid is equal for both legs. From herein, we include ALM, ALM/ht^2^, ALM/BMI, (E/I)_W,_ ALM/(E/I)_W_, LLM, and (E/I)_L_, LLM/(E/I)_L_ when referring to muscle measures.

### 2.4 Statistical analysis

We performed all data cleaning and analysis using SAS software version 9.4 (SAS Institute Inc., Cary, NC, USA). Frequencies, means, and standard deviations were calculated for categorical and continuous variables, respectively.

We conducted descriptive statistics and Spearman's ranked‐order correlation to assess bivariate correlations before transforming variables or the deletion of any outliers. We conducted a Shapiro–Wilks test of normality, examined skewness, and calculated kurtosis for each continuous variable. If the variable was non‐normally distributed based on the Shapiro–Wilks test at α = 0.05, the variable was transformed once and tested again for normality. If non‐normality remained, the variable was converted into a categorical variable, except for ALM/BMI to compare between other muscle measures. Univariate outliers were defined as having *Z*‐scores of greater than 3.29 and were then deleted from further analysis. Normality for each continuous variable was checked again once the univariate outliers were deleted. To remove the impact of multivariate outliers, we converted SAS software generated leverage from proc reg for all continuous variables of interest into Mahalanobis distances for each participant.^(^ ^29^ ^)^ We determined a participant to be a multivariate outlier if their Mahalanobis distance was greater than 27.88 based on a chi‐square distribution with an α = 0.001.^(^ ^30^ ^)^ After data cleaning, we tested the predictive abilities of traditional muscle mass measures (ALM, ALM/ht^2^, ALM/BMI), BIS‐derived fluid ratios ((E/I)_W_ and (E/I)_L_), and our novel muscle quality measures (ALM/(E/I)_W_ and LLM/(E/I)_L_) on handgrip strength and jump power using multiple linear regression with sex, age, race, BMI, and physical activity as covariates. We also tested for interactions between sex, race, and all muscle measures using multiple linear regression. Significance levels for the multiple linear regressions were set as α = 0.05.

Lastly, we tested the difference in model prediction effectiveness, or *R* from each multiple regression and subsequently *R* ^2^, between each of the muscle measures in predicting handgrip strength and jump power to determine if one muscle measure predicted handgrip strength or jump power best using Steiger's method. Steiger's method is used to compare how groups of different independent variables predict the same dependent variable based on the difference in *R* ^2^.^(^ ^31^ ^)^ We tested sequentially in order of lowest to highest *R* ^2^ values. Significance levels for model comparisons were adjusted with a Bonferroni correction because we compared six models to one another using Steiger's methods yielding an α = 0.008.^(^ ^32^ ^)^ We conducted post hoc power analyses for the multiple regression models for handgrip strength and jump power. Our power analyses suggest that all models had a power greater than 0.80. We used G*Power (version 3.1.9.7; Düsseldorf, Germany) for all power analyses.^(^ ^33^ ^)^

## 3 Results

Our cohort had 248 participants, of which 100 (40.3%) were Black and 144 (58.1%) were women. The average age and BMI of the sample were 49.25 years (SD ± 12.95 years) and 31.37 kg/m^2^ (SD ±7.97 kg/m^2^). Sixty‐one (24.6%) participants were between the ages of 25 and 40, while 58 (23.4%) were ages 41 to 50, 65 (26.2%) were ages 51 to 60, and 64 (25.8%) were ages 61 or older. Non‐sedentary adults comprised 147 (59.3%) of the sample. The averages of handgrip strength, jump power, ALM, ALM/ht^2^, ALM/BMI, ALM/(E/I)_W_, LLM, and LLM/(E/I)_L_ are detailed in Table 1. Technical challenges prevented 5 subjects from having valid LLM measurement; consequently, leg‐specific ECF and ICF data were unavailable. All continuous variables were natural log transformed except for handgrip strength, which was square root transformed to obtain a normal distribution.

**Table 1:** The Descriptive Statistics of Nontransformed Categorical and Continuous Variables

	Total (*N* = 248)		Female (*n* = 144)		Male (*n* = 104)	
	*n*	(%)	*n*	(%)	*n*	(%)
Race						
Black	100	(40.3)	68	(47.2)	32	(30.0)
White	148	(59.7)	76	(52.8)	72	(69.2)
Age (years)						
25–40	61	(24.6)	40	(27.8)	21	(20.2)
41–50	58	(23.4)	33	(22.9)	25	(24.0)
51–61	65	(26.2)	43	(29.9)	22	(21.2)
>61	64	(25.8)	28	(19.4)	36	(34.6)
Sedentariness						
METs = 0	101	(40.7)	59	(41.0)	42	(40.4)
METs >0	147	(59.3)	85	(59.0)	62	(59.6)

			
	Mean	(SD)	Mean	(SD)	Mean	(SD)
BMI (kg/ht ^2^ )	31.37	(7.97)	31.82	(8.01)	30.75	(7.92)
Age (years)	49.25	(12.95)	47.79	(12.50)	51.27	(13.34)
Handgrip strength (kg)	30.56	(11.85)	23.83	(7.52)	39.88	(10.36)
Jump power (kW)	2.62	(0.96)	2.17	(0.63)	3.24	(0.99)
ALM (kg)	23.89	(6.08)	20.82	(4.56)	28.15	(5.31)
ALM/ht ^2^ (kg/ht ^2)^	8.33	(1.99)	7.77	(1.55)	9.09	(2.26)
ALM/BMI (kg/(kg/ht ^2^ ))	0.78	(0.18)	0.67	(0.11)	0.94	(0.15)
ALM/(E/I)_W_ (kg)	62.67	(14.95)	53.88	(9.62)	74.85	(12.22)
LLM (kg)	17.77	(4.33)	15.84	(3.50)	20.45	(3.94)
LLM/(E/I)_L_ (kg) ^a^	6.7	(2.41)	5.91	(1.96)	7.97	(2.48)

METs = metabolic equivalent of task; BMI = body mass index; ALM = appendicular lean mass; ALM/ht^2^ = ALM divided by height squared; ALM/BMI = ALM divided by body mass index; (E/I)_W_ = BMI correct ratio of extracellular to intracellular fluid; LLM = leg lean mass; E/I_L_ = ratio of leg extracellular fluid to leg intracellular fluid.

^a^ *n* = 243 for LLM/(E/I)_L_.

Spearman's rank‐order analysis of bivariate correlations using the entire sample (*n* = 248) is presented in Table 2. Most importantly, handgrip strength was not associated with age (ρ = 0.006, *p* = 0.92), whereas jump power had a moderate negative association with age (ρ = −0.423, *p* < 0.001). ALM/ht^2^ was not associated with age (ρ = −0.083, *p* = 0.194), whereas ALM/BMI and ALM/(E/I)_W_ had slight negative associations with age (ρ = −0.140, *p* = 0.028; ρ = 0.163, *p* < 0.001, respectively). LLM/(E/I)_L_ had the greatest association with age out of all muscle measures with ALM included (ρ = −0.413, *p* < 0.001). The BIS‐derived ratios of (E/I)_W_ and (E/I)_L_ were also correlated with age with (E/I)_L_ most strongly associated with age (ρ = 0.247, *p* = <0.001; ρ = 0.559, *p* < 0.001, respectively).

**Table 2:** Spearman's Correlations Between Nontransformed Continuous Variables (*n* = 248)

	Age	BMI	HGS	JP	ALM	ALM/ht^2^	ALM/BMI	(E/I)_W_	ALM/(E/I)_W_	LLM	(E/I)_L_^A^	LLM/(E/I)_L_^A^
Age (years)		0.063	0.006	–0.423 ^c^	−0.099	−0.083	−0.140 ^b^	0.247 ^c^	−0.163 ^b^	−0.117	0.559 ^c^	−0.413 ^c^
BMI	0.063		−0.070	0.169 ^b^	0.538 ^c^	0.757 ^c^	−0.384 ^c^	0.904 ^c^	0.289 ^c^	0.577 ^c^	0.035	0.382 ^c^
HGS	0.006	−0.007		0.502 ^c^	0.500 ^c^	0.292 ^c^	0.607 ^c^	−0.177 ^b^	0.595 ^c^	0.44 ^c^	−0.162 ^b^	0.391 ^c^
JP	−0.423 ^c^	0.169 ^b^	0.502 ^c^		0.740 ^c^	0.599 ^c^	0.633 ^c^	−0.077	0.817 ^c^	0.717 ^c^	−0.548 ^c^	0.837 ^c^

BMI = body mass index; HGS = handgrip strength; JP = jump power; ALM = appendicular lean mass; ALM/ht^2^ = ALM divided by height squared; ALM/BMI = ALM divided by BMI; (E/I)_W_ = BMI correct ratio of extracellular to intracellular fluid; LLM = leg lean mass; (E/I)_L_ = ratio of leg extracellular fluid to leg intracellular fluid; α = 0.05.

^a^ *n* = 243 for LLM/(E/I)_L_.

^b^ *p* < 0.05.

^c^ *p* < 0.001.

We identified and removed three univariate outliers after transformations to obtain normality and found 0 multivariate outliers when testing Mahalanobis distances. This yielded *n* = 245 for all multivariate analyses. The multiple regression models show that each muscle measure was associated with handgrip strength (Table 3). Sex also consistently predicted handgrip strength across all models, with females having lower handgrip strength. Race was associated with handgrip strength in models with muscle measures, except for the LLM/(E/I)_L_ model. BMI was associated with handgrip strength among all muscle measure models largely with a greater BMI predicting lower handgrip strength. Conversely, age was not associated with handgrip strength in any model that included a muscle measure. Overall, the prediction models of covariates, all muscle measures, and BIS‐derived fluid ratios captured 46.5% to 51.4% of the variance in handgrip strength.

**Table 3:** Results of the Multiple Regression Models Showing the Association Between Handgrip Strength, a Covariate Only Model, and Muscle Measure (ALM/ht^2^, ALM/BMI, ALM/(E/I)_W_, LLM, and LLM/(E/I)_L_) Models With Covariates

Model	Intercept	Muscle measure	Sex	Age 40 to <50 years	Age 50 to <61 years	Age 61 years and older	Race	BMI	Non‐sed	*R* ^2^
Covariates only	7.829 ^d^		−1.452 ^d^	0.029	−0.187	−0.309 ^b^	−0.160	0.042	0.147	0.465
ALM	7.433 ^d^	0.820 ^d^	−0.749 ^d^	0.082	−0.060	−0.041	−0.240 ^b^	−1.320 ^d^	0.066	0.512
ALM/ht ^2^	7.696 ^d^	1.993 ^c^	−1.078 ^d^	0.077	−0.096	−0.160	−0.287 ^b^	−1.273 ^c^	0.090	0.488
ALM/BMI	5.259 ^d^	1.945 ^d^	−0.763 ^d^	0.096	−0.059	−0.048	−0.242 ^b^	0.644 ^b^	0.062	0.510
ALM/(E/I)_W_	1.454	1.937 ^d^	−0.726 ^d^	0.111	−0.013	0.026	−0.258 ^b^	−0.747 ^c^	0.051	0.514
LLM	6.180 ^d^	1.761 ^d^	−0.911 ^d^	0.098	−0.066	−0.063	−0.212 ^b^	−1.193 ^d^	0.066	0.505
LLM/(E/I)_L_^A^	7.910 ^d^	0.774 ^c^	−1.146 ^d^	0.144	−0.017	0.031	−0.199	−0.557 ^b^	0.067	0.491

BMI = body mass index; Non‐sed = non‐sedentary; ALM = appendicular lean mass; ALM/ht^2^ = ALM divided by height squared; ALM/BMI = ALM divided by BMI; (E/I)w = BMI correct ratio of extracellular to intracellular fluid; LLM = leg lean mass; (E/I)_L_ = ratio of leg extracellular fluid to leg intracellular fluid; α = 0.05.

Each muscle measure and BMI are log‐transformed (*n* = 245).

^a^ *n* = 243 for LLM/(E/I)_L_.

^b^ *p* < 0.05.

^c^ *p* < 0.01.

^d^ *p* < 0.001.

The multiple regression models predicting jump power also showed that each muscle measure was associated with jump power (Table 4). Unlike handgrip strength, sex was significantly associated with jump power in only the covariate ALM/ht^2^, LLM, and LLM/(E/I)_L_ models. Age was associated with jump power across all models in a dose‐respondent manner with greater age associated with lower jump power. Race was associated with jump power only in the ALM/ht^2^ model. BMI was associated with jump power in all muscle measure models except for the ALM/(E/I)_W_ models. No interaction terms between muscle measures and sex or muscle measures and race were significant when predicting jump power. The covariate model explained 60.2% of the variance in jump power. The addition of muscle measures to the covariate model increased the amount of variance in jump power explained, with (E/I)_W_ explaining 65.3%, (E/I)_W_ explaining 69.5%, ALM explaining 73.2%, ALM/ht^2^ explaining 71.2%, ALM/BMI explaining 73.8%, ALM/(E/I)_W_ explaining 75.9%, LLM explaining 72.2%, and LLM/(E/I)_L_ explaining 80.3% of the variance in jump power.

**Table 4:** Results of the Multiple Regression Models Showing the Association Between Jump Power, a Covariate Only Model, and Muscle Measure (ALM/ht^2^, ALM/BMI, ALM/(E/I)_W_, LLM, and LLM/(E/I)_L_) Models With Covariates

Model	Intercept	Muscle measure	Sex	Age 40 to <50 years	Age 50 to <61 years	Age 61 years and older	Race	BMI	Non‐sed	*R* ^2^
Covariates only	0.372		−0.478 ^d^	−0.127 ^c^	−0.239 ^d^	−0.491 ^d^	0.020	0.420 ^d^	0.069 ^b^	0.602
ALM	0.148	0.464 ^d^	−0.080	−0.098 ^c^	−0.167 ^d^	−0.340 ^d^	−0.026	−0.350 ^d^	0.023	0.732
ALM/ht ^2^	0.273	1.475 ^d^	−0.201 ^d^	−0.092 ^b^	−0.171 ^d^	−0.381 ^d^	−0.075 ^b^	−0.553 ^d^	0.027	0.712
ALM/BMI	−1.149 ^d^	1.150 ^d^	−0.070	−0.088 ^b^	−0.163 ^d^	−0.337 ^d^	−0.029	0.777 ^d^	0.019	0.738
ALM/(E/I)_W_	−3.491 ^d^	1.174 ^d^	−0.038	−0.078 ^b^	−0.133 ^d^	−0.288 ^d^	−0.040	−0.058	0.011	0.759
LLM	−0.599 ^c^	1.037 ^d^	−0.159 ^d^	−0.087 ^b^	−0.167 ^d^	−0.346 ^d^	−0.011	−0.307 ^d^	0.021	0.722
LLM/(E/I)_L_ ^a^	0.388 ^b^	0.717 ^d^	−0.190 ^d^	−0.039	−0.074 ^b^	−0.162 ^d^	0.000	−0.125 ^b^	−0.020	0.803

BMI = body mass index; Non‐sed = non‐sedentary; ALM = appendicular lean mass; ALM/ht^2^ = ALM divided by height squared; ALM/BMI = ALM divided by BMI; (E/I)_W_ = BMI correct ratio of extracellular to intracellular fluid; LLM = leg lean mass; (E/I)_L_ = ratio of leg extracellular fluid to leg intracellular fluid; α = 0.05.

Each muscle measure and BMI are log‐transformed (*n* = 245).

^a^ *n* = 243 for LLM/(E/I)_L_.

^b^ *p* < 0.05.

^c^ *p* < 0.01.

^d^ *p* < 0.001.

When we used Steiger's test to compare handgrip strength model differences in *R* ^2^, no significant differences were found between the covariate model and models with the addition of ALM, ALM/ht^2^, ALM/BMI, LLM, ALM/(E/I)_W_, or LLM/(E/I)_L_ (covariate model: versus ALM *p* = 0.293; versus ALM/ht^2^ *p* = 0.615; versus ALM/BMI *p* = 0.319; versus ALM/(E/I)_W_ *p* = 0.293; versus LLM *p* = 0.380; versus LLM/(E/I)_L_ *p* = 0.584). No significant differences in handgrip strength models between ALM/(E/I)_W_ and ALM/ht^2^, ALM/(E/I)_W_ and ALM/BMI, or ALM/(E/I)_W_ and LLM/(E/I)_L_ were found (*p* = 0.673, *p* = 0.914, and *p* = 0.614, respectively).

**Fig 1:**
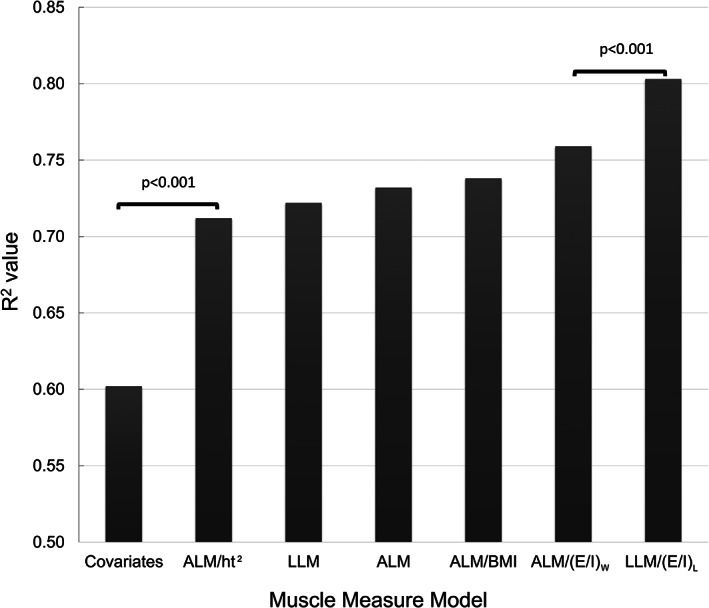
The results from Steiger's model comparison test. When comparing *R* ^2^ values between models, each muscle measure model explained significantly higher variance in jump power than the covariate model. Among the muscle measure models, appendicular lean mass (ALM) adjusted for height squared (ALM/ht^2^), leg lean mass (LLM), ALM, ALM adjusted for body mass index (ALM/BMI), and ALM adjusted for the BMI corrected ratio of extracellular to intracellular fluid (ALM/(E/I)W) were not significantly different in the amount of jump power variance they explained when significance levels were adjusted using a Bonferroni correction. LLM/(E/I)L significantly explained the highest amount of variance in jump power with an *R* ^2^ value of 0.803. Brackets indicate comparisons between models with *p* values of those comparison indicated on each bracket.

In contrast, the Steiger's test for jump power showed significant model differences in *R* ^2^ for all muscle measures, which explained more variance in jump power than the covariate model alone (*p* < 0.001). When comparing ALM, ALM/ht^2^, ALM/BMI, LLM, and ALM/(E/I)_W_, only ALM/ht^2^ and ALM/(E/I)_W_ significantly differed with ALM/(E/I)_W_, explaining more jump power variance than ALM/ht^2^ when α = 0.05; however, the difference in *R* ^2^ between ALM/ht^2^ and ALM/(E/I)_W_ was no longer significant after the Bonferroni correction of α = 0.008 for multiple comparisons. Our new measure of LLM/(E/I)_L_ significantly explained the most variance in jump power and differed from ALM/(E/I)_W_ after the Bonferroni correction (*p* < 0.001; Fig. 1).

## 4 Discussion

This study introduces LLM/(E/I)_L_, which outperformed all traditional measures of muscle. In this cohort, LLM/(E/I)_L_, which includes a surrogate of muscle quality, predicts jump power better than ALM/ht^2^, ALM/BMI, and ALM/(E/I)_W_. ALM/(E/I)_W_ predicted a large amount of variation in jump power but did not significantly outperform ALM/ht^2^ or ALM/BMI when adjusted for multiple comparisons. The normalization of LLM or ALM by the ratio of extracellular to intracellular fluid adjusts for fluid distribution changes found with aging, which is not accounted for by either ALM/ht^2^ or ALM/BMI. We suggest that these novel measures be utilized in larger and more diverse studies. If our results are validated, the BIS adjustment of ALM should receive consideration of inclusion as a component of future sarcopenia diagnostic parameters.

Our results demonstrate that these novel muscle measures, which incorporate both muscle mass and muscle quality, outperform the prediction of muscle function based on traditional muscle measures that assess mass alone. Although no consensus definition of muscle quality exists, working definitions include contractile area, fat infiltration, and in this present study, fluid volume distribution.^(^ ^15^, ^16^, ^17^, ^18^ ^)^ Regardless, muscle quality predicts muscle function, recovery, and mortality, yet clinicians do not currently assess quality attributes.^(^ ^18^, ^34^, ^35^, ^36^ ^)^ Although ambiguous, muscle quality provides an explanation for the large drop in muscle function with age that cannot be explained by the loss of muscle mass alone.^(^ ^15^, ^37^ ^)^ Incorporating muscle quality into the definition of sarcopenia may improve the utility of its diagnosis. The characterization of muscle quality may also identify those individuals with adequate muscle mass but low muscle quality. Reduction in muscle quality likely occurs before any appreciable loss of mass can be measured, which also supports the higher association of muscle quality with age.^(^ ^15^ ^)^ Utilizing a muscle‐quality measure may then serve as an early detection of sarcopenia and provide an opportunity for timely interventions needed to mitigate consequential outcomes. Our results support the use of combining DXA and BIS for two improved measures of muscle, LLM/(E/I)_L_ and LLM/(E/I)_L_, for predicting muscle function.

When examining the statistical comparisons of muscle measures, handgrip strength is not strongly predicted by ALM, ALM/ht^2^, ALM/BMI, (E/I)_W_, ALM/(E/I)_W_, LLM, (E/I)_L_, or LLM/(E/I)_L_ in the present study. Although all muscle measures seem to predict jump power, our results do not suggest these muscle measures are meaningfully associated with handgrip strength. Our multivariate models, using any of our muscle measures predicting handgrip strength, explained only 2.3% to 4.9% more than the multivariate model, which included only covariates. This suggests the muscle measures only added a clinically irrelevant amount of explanation of handgrip strength.

In contrast, our results suggest jump power is better associated with age and instrumental muscle measures in bivariate and multivariate models. This supports the use of jump power as an alternative, more appropriate method to determine muscle function than handgrip strength, especially in relation to age.^(^ ^19^, ^20^, ^21^ ^)^ Assessing jump power requires both legs and coordinated movement, which can be seen as an indicator of muscle motor unit function and electrophysiological health, instead of only handedness or individual muscle groups in handgrip strength.^(^ ^38^ ^)^ Indeed, lower leg strength is associated with falls and fractures, both of which are outcomes of sarcopenia.^(^ ^22^, ^23^ ^)^

In all our multivariate models, categories of increasing age negatively impacted jump power, providing evidence jump power is negatively associated with age in an expected physiological way. Because jump power is significantly associated with age in a dose‐respondent manner across all muscle measures, LLM/(E/I)_L_ is associated with age, and LLM/(E/I)_L_ best predicts jump power, we recommend jump power in conjunction with LLM/(E/I)_L_ as appropriate diagnostic measures for sarcopenia diagnosis. In addition, because the ALM/(E/I)_W_ model explained a large amount of variation in jump power and did not have sex or BMI as significant covariates, this novel measure may also lead to a more clinically useful approach of measuring muscle health.

Although these results are promising, this study does have several limitations. Our study population included only participants who identified as Black or White, limiting its applicability in other races/ethnicities, which do show average differences in skeletal muscle mass and differences in skeletal muscle mass decline.^(^ ^39^ ^)^ Our cross‐sectional study design is not able to use any muscle mass or quality measure to prospectively predict health outcomes, and at this point, the associations are based on correlations. Being non‐sedentary is likely not a predictor in many of our models because we used a dichotomous variable indicating “yes or no” for being non‐sedentary because of the lack of individuals who reported any METS, which is not a real‐world scenario as the lowest level of METS measure oxygen consumption levels in a sedentary state.^(^ ^40^ ^)^ Other studies have shown that increased resistance training increases muscle mass and muscle quality,^(^ ^41^, ^42^, ^43^, ^44^, ^45^ ^)^ suggesting physical activity may be a modifier in the relationship between these muscle measures and muscle function. Because of the non‐normal distributions, we used transformations of each continuous variable, which make the interpretability of results difficult. Our transformations did not produce a normal distribution for ALM/BMI, which had a tendency toward a bimodal distribution, although non‐normality was slightly significant based on the Shapiro–Wilk test (*p* = 0.018). Our novel method also requires both DXA and BIS versus the current diagnosis of sarcopenia using DXA to diagnose muscle mass, which may limit the applicability of our method in a clinical setting.

However, our study does have substantial strengths, including the large number of individuals who completed both DXA and BIS and jump power. Other studies that have assessed BIS and DXA have had limited sample numbers.^(^ ^11^, ^16^, ^46^ ^)^ The present study also includes Black and White individuals aged 25 to 75 years with variations in BMI and age expanding upon results from our initial study examining the potential of ALM/(E/I)_W_ conducted within an older White population.^(^ ^16^ ^)^ In our present study, we show jump power is associated with age regardless of the muscle measure across various ages, including those who would not be traditionally targeted for sarcopenia interventions. We show ALM/(E/I)_W_ may be unbiased when assessing muscle between sexes, which would be advantageous in a clinical setting, although LLM/(E/I)_L_ does not account for sex. Both ALM/(E/I)_W_ and LLM/(E/I)_L_ are not affected by race, although more research is needed to expand ALM/(E/I)_W_ and LLM/(E/I)_L_ into more racially and ethnically diverse populations. In addition, our results suggest our measure of LLM/(E/I)_L_ is clinically relevant because of the high *R* ^2^ value when predicting jump power using a combination of two noninvasive techniques.

Future studies should use ALM/(E/I)_W_ and LLM/(E/I)_L_ to predict health outcomes including falls, fractures, disability, quality of life, and mortality. Research examining the associations between ALM/(E/I)_W_ and LLM/(E/I)_L_ and chronic diseases, such as diabetes or congestive heart failure, may also support the use of our novel muscle measure predictors of chronic disease severity. Muscle function and muscle quality are consistently predictors of mortality, although muscle mass is only an occasional predictor of mortality;^47^, ^48^, ^49^, ^50^ ^)^ however, ALM/(E/I)_W_ and LLM/(E/I)_L_ remain to be tested as a predictor of mortality. Longitudinal studies will elucidate the connection between the changes in muscle quality and muscle function. Studies with higher sample sizes and more diverse study populations will be able to explore more minute differences in health outcomes between muscle quality and muscle mass, leading to better evidence to define sarcopenia.

Our novel measure, LLM/(E/I)_L_, outperformed traditional sarcopenia diagnosis measures, specifically ALM/ht^2^ and ALM/BMI, when predicting jump power. Additionally, we previously observed ALM/(E/I)_W_ outperformed ALM/ht^2^ in an older White population when predicting muscle function, and now have demonstrated expanded utility in a larger, more age and racially diverse population. Future research into the predictive capabilities of ALM/(E/I)_W_ and LLM/(E/I)_L_ on health outcomes, including falls and fractures, among large samples is necessary. Our muscle measures are improved measures of muscle and should be considered for the identification of sarcopenia.

## Disclosures

All authors state that they have no conflicts of interest.

5

### Peer review

The peer review history for this article is available at https://publons.com/publon/10.1002/jbm4.10527.
